# The Efficacy of Venlafaxine, Flunarizine, and Valproic Acid in the Prophylaxis of Vestibular Migraine

**DOI:** 10.3389/fneur.2017.00524

**Published:** 2017-10-11

**Authors:** Fenye Liu, Tianbao Ma, Xiaolin Che, Qirong Wang, Shudong Yu

**Affiliations:** ^1^Department of Traditional Chinese Medicine, Shandong Provincial Hospital Affiliated to Shandong University, Jinan, China; ^2^Department of Hearing Central, Women and Children’s Health Care Hospital of Linyi, Linyi, China; ^3^Department of Otolaryngology, Shandong Qianfoshan Hospital, Jinan, China

**Keywords:** vestibular migraine, prophylaxis, efficacy, venlafaxine, flunarizine, valproic acid

## Abstract

**Background:**

Different types of medications are currently used in vestibular migraine (VM) prophylaxis, although recommendations for use are generally based on expert opinion rather than on solid data from randomized trials. We evaluated the efficacy and safety of venlafaxine, flunarizine, and valproic acid in a randomized comparison trial for VM prophylaxis.

**Methods:**

Subjects were randomly allocated to one of three groups (venlafaxine group, flunarizine group, and valproic acid group). To assess the efficacy of treatment on vertigo symptoms, the following parameters were assessed at baseline and 3 months after treatment: Dizziness Handicap Inventory (DHI) scores, number of vertiginous attacks in the previous month, and Vertigo Severity Score (VSS). Adverse events also were evaluated.

**Results:**

A decrease in DHI total scores was shown following treatment with all three medications, with no obvious differences between the groups. Treatment effects differed, however, in the DHI physical, functional, and emotional domains with only venlafaxine showing a decreased effect in all of three domains. Flunarizine and valproic acid showed an effect in only one DHI domain. Venlafaxine and flunarizine showed decreased VSS scores (*p* = 0 and *p* = 0.03, respectively). Although valproic acid had no obvious effect on VSS (*p* = 0.27), decreased vertigo attack frequency was observed in this group (*p* = 0). Venlafaxine also had an effect on vertigo attack frequency (*p* = 0), but flunarizine had no obvious effect (*p* = 0.06). No serious adverse events were reported in the three groups.

**Conclusion:**

Our data confirm the efficacy and safety of venlafaxine, flunarizine, and valproic acid in the prophylaxis of VM, venlafaxine had an advantage in terms of emotional domains. Venlafaxine and valproic acid also were shown to be preferable to flunarizine in decreasing the number of vertiginous attacks, but valproic acid was shown to be less effective than venlafaxine and flunarizine to decrease vertigo severity.

**Trial registration:**

ChiCTR-OPC-17011266 (http://www.chictr.org.cn/).

## Introduction

Migraine and vertigo are among the most common health concerns in the general population. The link between migraine and vertigo was described for the first time by Aretaeus of Cappadocia in 131 BC. Nowadays, vestibular migraine (VM) is considered a distinct diagnostic entity by both the Barany Society and the International Headache Society (1–3), and VM is considered one of the most common vestibular disorders in the general population with a lifetime prevalence of 1% and 1-year prevalence of 0.9% (4).

Vestibular migraine presents with attacks of vertigo or dizziness lasting several minutes to 3 days. Vertigo can precede the migraine attack but can also occur during or after the headache (5, 6). Vertigo can also occur in the absence of a typical migrainous headache, and symptoms such as phono-/photophobia, osmophobia, nausea, and vomiting, and aggravation by movement can present in some patients. Currently, there are no biological markers for the diagnosis of VM. Moreover, VM vertigo shares features with some other clinical conditions (7). Taken together, these factors can present diagnostic difficulties.

Since the mechanisms underlying VM remain insufficiently known, different types of medications are typically used in VM prophylaxis including β-blockers (propranolol or metoprolol), calcium overload blockers (flunarizine), anticonvulsants (valproic acid or topiramate), and antidepressants (amitriptyline and venlafaxine) (8–11). Unfortunately, current treatment recommendations are based on expert opinion rather than on solid data from randomized trials. Few studies to date have evaluated the effectiveness of venlafaxine therapy in VM prophylaxis (12). In this randomized comparison trial study, the efficacy and safety of venlafaxine, flunarizine, and valproic acid in VM prophylaxis were investigated.

## Methods

### Patients

**Figure 1:**
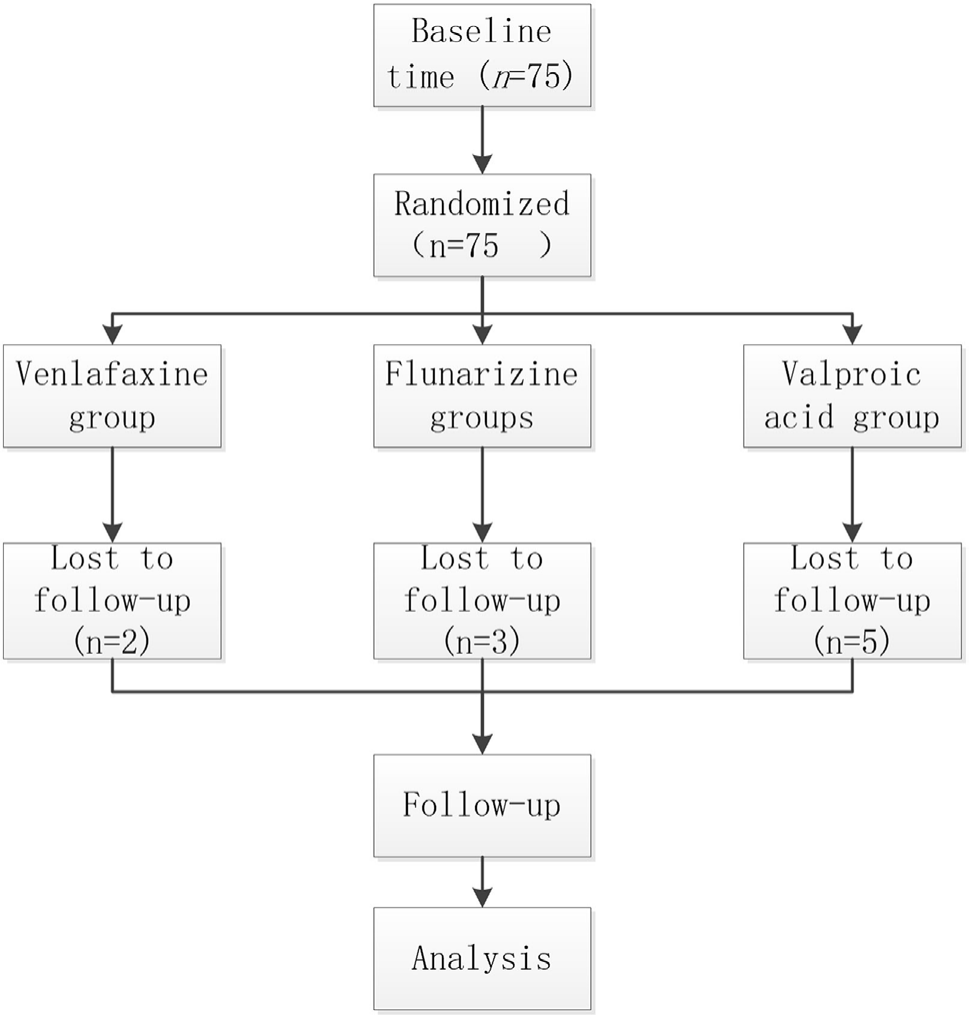
CONSORT flow diagram of patient disposition.

A single-blinded randomized comparison trial of 3 months duration was carried out in Shandong Qianfoshan Hospital according to a protocol approved by the hospital ethics committee. Patients were enrolled between January 1, 2016 and December 25, 2016. An ear, nose, throat, and neurotological examination was performed followed by specific audiovestibular investigations and imaging when required. Eligible participants included male and female outpatients aged 18 years or older with a primary diagnosis of VM [confirmed VM or probable VM (pVM)] based on standard criteria (Table 1) (1–3). Additional inclusion criteria were two or more migraine attacks per month or very disabling attacks and no improvement observed following non-pharmaceutical treatment. Patients with associated benign paroxysmal positional vertigo, Meniere’s disease, chronic discharging ear, history of ear surgery, profound hearing loss, women who were breastfeeding, and patients with major cardiovascular, metabolic, gastrointestinal, and neurologic diseases were excluded from the study. Subjects were randomly allocated to one of three groups [venlafaxine group (VG), flunarizine group, and valproic acid group (VAG)] by the order of visits. The patients were unaware of the medication that they took throughout the trial. To assess the efficacy of treatment on vertigo symptoms, the following parameters were assessed at baseline and 3 months after treatment: Dizziness Handicap Inventory (DHI) scores (13), number of vertiginous attacks in the previous month, and Vertigo Severity Score (VSS). These primary outcome measure methods were similar to those recommended for migraine (14). The DHI is a validated, self-reported questionnaire designed to evaluate the precipitating physical factors associated with dizziness and unsteadiness as well as the functional and emotional consequences of vestibular disease. VSS is a visual analog scale with 0 indicating the absence of vertigo and 10 representing extremely severe vertigo. Adverse events related to study medication were assessed during each visit (Figure 1).

**Table 1:** Diagnostic criteria for definite and probable VM (pVM).

Definite VM At least 5 episodes with vestibular symptoms of moderate or severe intensity, lasting 5 min to 72 h Current or previous history of migraine with or without aura according to the International Classification of Headache Disorders (ICHD) One or more migraine features with at least 50% of the vestibular episodes: – headache with at least two of the following characteristics: one sided location, pulsating quality, moderate or severe pain intensity, aggravation by routine physical activity – photophobia and phonophobia – visual aura Not better accounted for by another vestibular or ICHD diagnosis pVM At least 5 episodes with vestibular symptoms of moderate or severe intensity, lasting 5 min to 72 h Only one of the criteria B and C for vestibular migraine is fulfilled (migraine history or migraine features during the episode) Not better accounted for by another vestibular or ICHD diagnosis

*Vestibular symptoms include the following: spontaneous vertigo, positional vertigo, visually induced vertigo, head motion-induced vertigo, and head motion-induced dizziness with nausea. Vestibular symptoms are “moderate” if they interfere with but do not prohibit daily activities and “severe” if patients cannot continue daily activities. Adapted from Lempert et al. (1–3)*.

### Treatment

Patients in the VG received 25 mg venlafaxine (Efexor XR, 75 mg, Wyeth, USA) at bedtime for 6 days, followed to a total daily dose of 37.5 mg (low than normal dosage). Patients in the flunarizine group received 10 mg flunarizine (Sibelium, 5 mg, Xian Janssen, China) at bedtime for the entire treatment period. Patients in the VAG received valproic acid (Depakin, 500 mg, Sanofi, France) at a dose of 1,000 mg/day (twice daily) for the entire treatment period.

### Statistical Analysis

Data were analyzed using the SPSS version 19 statistical package (IBM Corporation, New York City, NY, USA). Baseline demographics and clinical characteristics were compared using the ANOVA test for numeric data (age) and chi-square tests for nominal data (sex and diagnosis). Paired *t*-tests were used to compare VSS, DHI, and attack frequency before and after treatment. ANOVA tests were used to compare changes in DHI scores from baseline to the final visit. Significance was set at *p* < 0.05 for all statistical tests.

## Results

### Patients

A total of 75 subjects with either confirmed VM or pVM were recruited into this study and were treated with venlafaxine, flunarizine, or valproic acid for 3 months. Five patients in the VAG (unexpected improvement, three patients; adverse events, two patients), two patients in the venlafaxine group (consent withdrawn), and three patients in the flunarizine group (lost to follow-up, two patients; consent withdrawn, one patient) terminated the study prematurely (Figure 1). Data on the age, gender, and diagnosis in the different groups are shown in Table 2. There were no conspicuous differences between three groups at baseline. Data on the VSS, DHI, and attack frequency in the different groups before treatment are shown in Table 3; no conspicuous differences were observed between the three groups (*p* > 0.05).

**Table 2:** Demographic profile of patients in the study.

	Venlafaxine	Flunarizine	Valproic acid	*p*-Value
**Sex**				
Male	7	8	5	
Female	16	14	15	0.727
Total	23	22	20	
**Diagnosis**				
dVM	6	8	6	0.754
pVM	17	14	14	
**Age (years)**				
$\left(\bar{x}\pm S\right)$	53.22 ± 15.55	51.45 ± 15.43	52.35 ± 16.01	0.931

*dVM, definite VM; pVM, probable VM*.

**Table 3:** Groups before and after treatment with regard to symptom (VSS), disability (DHI), and attack frequency.

	Venlafaxine			Flunarizine			Valproic acid		
	Before	After	*p*	Before	After	*p*	Before	After	*p*
VSS	5.96 (1.72)	3.78 (1.28)	0	6.41 (1.99)	5.86 (1.55)	0.03	5.8 (1.82)	5.3 (1.08)	0.27
DHI-e	14.17 (6.95)	9.39 (5.03)	0	16.91 (6.84)	15.45 (7.07)	0.115	16.8 (4.7)	15.3 (4.91)	0.11
DHI-f	14.87 (7.11)	11.57 (5.36)	0.01	16.64 (5.57)	13.45 (6.24)	0.013	16.60 (5.20)	13.2 (4.96)	0.02
DHI-p	12.61 (4.80)	9.91 (5.24)	0.018	13.00 (4.44)	10.91 (4.69)	0.041	13.4 (4.45)	10.30 (4.91)	0.01
DHI-t	41.74 (16.90)	31.3 (14.14)	0.001	46.64 (15.15)	39.82 (16.35)	0.019	46.80 (13.45)	38.7 (13.58)	0.02
Frequency	5.83 (3.2)	3.09 (1.68)	0	4.95 (3.28)	4.15 (2.46)	0.057	5.1 (3.14)	2.35 (1.79)	0

*Values are expressed as the median (SD)*.

*VSS, Vertigo Severity Score; DHI-t, Dizziness Handicap Inventory-total score; e, emotional; f, functional; p, physical; DHI, Dizziness Handicap Inventory*.

### Efficacy

**Figure 2:**
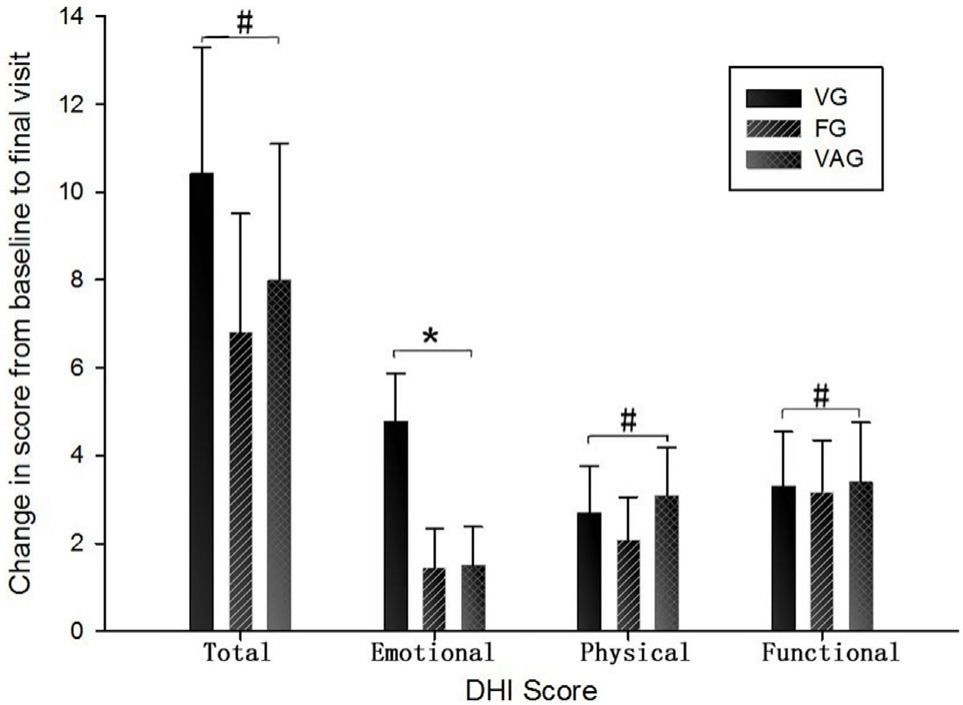
Change in DHI scores from baseline to final visit for three medications $\left(\bar{\text{x}}\pm \text{SE}\right)$. DHI, Dizziness Handicap Inventory; VG, venlafaxine group; FG, flunarizine groups; VAG, valproic acid group (^#^*p* > 0.05; *p < 0.05).

Changes in the different outcome measures in both groups are shown in Table 3 and Figure 2. Significantly decreased total DHI scores were observed following treatment with all three medications (*p* < 0.05, Table 3) with no obvious differences between three groups (*p* > 0.05, Figure 2). However, treatment effects differed according to the DHI physical, functional, and emotional domains. The emotional domains of DHI were significantly decreased following treatment with venlafaxine (*p* = 0), while no difference was observed in the flunarizine and VAGs (*p* = 0.12 and *p* = 0.11, respectively). A significant difference was observed between the three groups (*p* < 0.05, Figure 2). For the physical and functional domains of DHI, a significant decrease was observed after treatment with all three medications (*p* < 0.05) although there were no differences between the groups (*p* > 0.05, Figure 2). Vertigo severity scoring on VSS was inconsistent after treatment in the three groups, although venlafaxine and flunarizine showed decreased VSS scores (*p* = 0 and *p* = 0.03, respectively). Although valproic acid had no obvious effect on VSS (*p* = 0.27), decreased vertigo attack frequency was observed in this group (*p* = 0). Venlafaxine had an effect on vertigo attack frequency (*p* = 0), but flunarizine had no obvious effect (*p* = 0.06).

### Safety

No serious side effects were observed in patients who completed the study period. In the VG, two patients suffered from nausea, one suffered from insomnia, one suffered from palpitation, and one complained of lethargy. In the VAG, two patients reported nausea, one reported somnolence, and one reported indigestion. In the flunarizine group, five patients reported somnolence and one patient reported nausea.

## Discussion

Prophylactic treatment of VM is mainly intended to reduce attack frequency, duration, and severity of vertigo. Among the many types of medications prescribed for migraine prophylaxis, valproic acid, venlafaxine, and flunarizine are the most representative. In this randomized comparison trial, the efficacy and safety of these three medications were investigated for prophylaxis in patients diagnosed with VM. Our findings suggest that the medications were safe and effective in reducing DHI total score but had difference effects in the domains of VSS score and attack frequency; venlafaxine had an advantage in terms of emotional domains. Venlafaxine and valproic acid also were shown to be preferable to flunarizine in decreasing the number of vertiginous attacks, but valproic acid was shown to be less effective than venlafaxine and flunarizine to decrease vertigo severity.

Although several studies have reported the efficacy of venlafaxine in the prophylaxis of migraine (15–17), very little data exist on its efficacy for VM prophylaxis (12) although some studies have investigated flunarizine (16, 18) and valproic acid for VM prophylaxis (9, 19). In this study, all three medications had effects on VM. Valproic acid had no effect on the severity of vertigo although it significantly reduced the number of vertigo episodes. Valproic acid is an anticonvulsant medication, and its mechanism of migraine prophylaxis may be related to ion channel blocking (20). Flunarizine is also a channel blocker, selectively blocking calcium channels. Therefore, ion channels may have a role in the occurrence of VM; calcium channels may affect the severity of vertigo while other ion channels that can be blocked by valproic acid may affect attack frequency.

Venlafaxine, a serotonin–norepinephrine reuptake inhibitor, is a clinically effective and safe medication that is widely used to treat depression. In recent years, it has been shown to have potential in migraine prophylaxis (15–17). Similar to its use in the treatment of migraine, venlafaxine is a safe and effective medication for the prophylaxis of VM. It is well known that the link between vertigo and anxiety, VM patients were more anxious than migraine patients without vestibular symptoms (21, 22). As a medication to treat anxiety, it has obvious effects on emotional symptom relief that differ from the other medications investigated in the current study. For this reason, venlafaxine has a better response in VM patients, especially those with anxiety (23). In our study, venlafaxine can also decrease vertigo attack frequency and severity. We believe that venlafaxine confers some advantages over other medications for VM prophylaxis through several factors. Similar to the onset of migraine, 5-HT levels affect the onset of vestibular symptoms (24, 25). Venlafaxine decreases 5-HT levels and subsequently vertigo attack frequency and can also reduce the levels of certain inflammatory cytokines (26–28) that play a role in episodic vertigo. Venlafaxine also exerts neuroprotection effects (29) that may decrease the severity of vertigo.

It should be noted that the dose of venlafaxine used in our study was lower than that reported previously (12). In our preliminary study, where we found that a daily dose of 37.5 mg had similar effects to the maximum daily dose of 75 mg. Therefore, we used 37.5 mg as the maximum daily dose in this study to further reduce side effects. In this study, none of the three medications was associated with serious side effects, and so was considered safe at the investigated doses. It also should be noted that most of the patients in our study were pVM. The reason is that pVM has higher morbidity than definite VM.

The results of this study should be interpreted within its limitations. This study had no placebo arm because the major purpose was to investigate the efficacy of three recommendatory medications (10, 12, 18, 30). But that does not allow conclusions to be drawn on the absolute efficacy of the medications. Second, because both drugs showed beneficial effects and the effects depended on subjective experience or feeling (the outcome measures used mostly based on patients’ feeling), it was unclear whether the results were placebo effects. The low patient numbers may also have provided less power to detect between-group differences. Furthermore, the efficacy of the three medications was examined for only a short period of time. Future long-term studies with a larger sample size and placebo arm are therefore warranted.

## Conclusion

Our data confirm the efficacy and safety of venlafaxine, flunarizine, and valproic acid in the prophylaxis of VM. Venlafaxine had an advantage in terms of emotional domains. Venlafaxine and valproic acid also were shown to be preferable to flunarizine in decreasing the number of vertiginous attacks, but valproic acid was shown to be less effective than venlafaxine and flunarizine to decrease vertigo severity.

## Ethics Statement

The study was approved by the ethics committee of Shandong Qianfoshan Hospital. All patients provided written informed consent.

## Author Contributions

SY conceived and designed the study. FL, TM, and XC performed the experiments. FL wrote the paper. QW reviewed and edited the manuscript. All the authors read and approved the manuscript.

## Conflict of Interest Statement

The authors declare that they have no financial and personal relationships with other people or organizations that can inappropriately influence our work, and there is no professional or other personal interest of any nature or kind in any product, service, and/or company that could be construed as influencing the position presented in, or the review of, the manuscript entitled.
